# Stricture of the Urethra

**Published:** 1852-01

**Authors:** 


*Stricture of the Urethra, its Pathology and Treatment, comprising Observations on the Curative Powers of the Potassa Fusa in that Disease; with Cases.* By Robert Wade, F. R. C. S. &c., 2d Edition. Churchill, London, pp. 265.

As far as we know, John Hunter, the great originator of so many beneficent improvements in the treatment of surgical diseases, was the first to employ caustics in the treatment of stricture of the urethra. His first experimental applications were very rude, producing considerable imflammatory action along the course of the urethra; soon, however, his ingenuity suggested an instrument for the due application of the agent he wished to employ, and he continued to use it when occasions afforded. His pupil, Sir. E. Home, continued and improved upon his practice, using principally, if not solely, the nitrate of silver as his agent, at the same time a contemporary, Mr. Whately, used with great success the potassa fusa in very minute quantities, and without any ill effects. For some time the use of caustics to the strictured urethra flourished; but from some cause, (whether it was that the practice was abused we know not,) it was condemned and abandoned by the great surgeons of the last generation, both in England and France. Recently, thanks to the persevering energy and skillful management of Mr. Wade, the practice has revived, and in his hands, especially, has been pursued with a success which claims the thoughtful attention of every surgeon, when called upon to treat a hard cartilaginous stricture, which does not yield to treatment by bougies alone, or those irritable ones which so frequently, from the spasmodic action, induced during the passage of the bougie, are extremely difficult to manage, most especially, however, does Mr. Wade urge the treatment to potassa fusa in cases of impervious stricture, preferring that plan of treatment, and as we think, very justly to the much discussed operation of cutting into the urethra from the perineum, practiced by Mr. Syme, and followed by a few other surgeons, with very questionable results. The reader will find Mr. Wade's opinions, with regard to this operation, fully developed at the end of the book. Of the other methods employed in the treatment of severe strictures, we have no space to say more than that the stilette catheter of Stafford has still its advocates, although we believe that our hospitals can produce but few cases in which it has been employed of late years; its advantages are all attainable by the use of potassa fusa, whilst its disadvantages are avoided. The forcible rupture of the strictured portion of the urethra has also been revised of late, and may be performed by means of instruments invented by Mr. Thomas Wakley, with ease, and we believe safety, although from the limited time these instruments have been before the profession it would be unwise, at present, to do more than recommend a fair trial of them. We ourselves, however, have seen them used by the inventor with marked success. Returning to our author, we cannot too highly applaud the clear and practical manner, in which he has described the cases, which will be beneficial by the potassa fusa, the method of its application, and the reasons he entertains for preferring this caustic to nitrate of silver, or any other, as well as to all other modes of treatment. We can confidently say, that there is nothing wanting in this treatise on stricture—no untoward event which is not anticipated, no species of stricture which is not discussed, and no kind of treatment which is not analyzed; whilst the principal feature, the reintroduction to practice of the potassa fusa, in an efficient and safe and practical manner, is worked out in the simplest, yet most comprehensive language. There is no bigotry in this volume; with a high and justifiable confidence in this plan of treatment, Mr. Wade rides no hobby, but gives due honor to, and himself employs the simple bougie and other modes of treatment with discriminating judgment. Many cases are given illustrative of the opinions put forth in the work, and of the practice pursued by Mr. Wade; from the perusal of which, those practitioners especially who have not the advantage of seeing hospital practice in large towns, will derive real practical information.

